# Estimating pediatric lung volumetric parameters via rapid, limited‐slice, free‐breathing thoracic dynamic MRI

**DOI:** 10.1002/mp.70628

**Published:** 2026-08-06

**Authors:** You Hao, Yubing Tong, Caiyun Wu, Joseph M. McDonough, Samantha Gogel, David M. Biko, Patrick J. Cahill, Jason B. Anari, Drew A. Torigian, Jayaram K. Udupa

**Affiliations:** ^1^ Department of Radiology University of Pennsylvania Philadelphia Pennsylvania USA; ^2^ Center for Thoracic Insufficiency Syndrome Children's Hospital of Philadelphia Philadelphia Pennsylvania USA; ^3^ Department of Radiology Children's Hospital of Philadelphia Philadelphia Pennsylvania USA; ^4^ Department of Radiology Hospital of the University of Pennsylvania Philadelphia Pennsylvania USA

**Keywords:** dynamic magnetic resonance imaging (dMRI), lung volume, pediatric lung imaging, spatio‐temporal sampling, thoracic insufficiency syndrome (TIS)

## Abstract

**Background:**

Dynamic, separate lung volume assessment during breathing is critical for evaluating thoracic disorders like scoliosis, thoracic insufficiency syndrome (TIS), and pulmonary diseases, as well as for monitoring treatments. While traditional volumetric assessment relies on static 3D imaging during breath‐holds, this is unfeasible for many pediatric patients or those with severe respiratory distress. Free‐breathing 4D dynamic MRI (dMRI) is the ideal modality due to its lack of radiation, excellent soft‐tissue contrast, and flexible imaging planes. However, its clinical use is hampered by long acquisition times, during which patients struggle to maintain stable, consistent breathing patterns or remain still. Consequently, methods to accelerate dMRI acquisition while maintaining volumetric accuracy are urgently needed to make free‐breathing assessments clinically practical.

**Purpose:**

We present an observational study involving free‐breathing short‐scan‐time dynamic MRI (dMRI) method that can be routinely used for computing dynamic lung volumes accurately.

**Methods:**

(1) Full sampled free‐breathing sagittal 2D dMRI scans are gathered from 45 normal children via bSSFP sequence. Sparse dMRI (s‐dMRI) scans are simulated from these datasets by optimally subsampling in the spatio‐temporal domains via a limited number of selected sagittal locations and time instances. (2) A 4D image is constructed from both scans. Lungs are segmented from 4D image, and their volumes from full and sparse dMRI scans are computed. (3) A regression model is developed to predict full‐scan volumes from sparse‐scan data on a training set. (4) The accuracy is analyzed on both synthesized sparse dMRI scans from a separate fully‐sampled‐scan test set and actual s‐dMRI scans prospectively acquired from 10 normal children.

**Results:**

With 5 slices per lung and 40 time points, the predicted volume showed a ∼2% deviation from the full‐scan volume, with a total scan‐time of ∼9 min (vs. 49.72 ± 5.01 min for the full scan with 15–22 slices per lung and 80 time points). When the spatial sampling was increased to full number of slices (15‐22 per lung) but only 40 time points, these metrics become 0.4%, and 24.86 ± 2.5 min.

**Conclusions:**

s‐dMRI is a practical approach for computing dynamic lung volumes that can be used routinely with no radiation concern, especially on patients who cannot tolerate long acquisition times.

## 1 INTRODUCTION

Assessment of dynamic volumes of the two lungs separately during tidal breathing is useful for assessment of many pediatric and adult thoracic disorders^1^ such as scoliosis, thoracic insufficiency syndrome (TIS),^2^, ^3^ and various pulmonary disorders,^4^ and for determining the effects of therapeutic interventions.^5^ Several imaging modalities have been used for lung volume estimation, including chest radiography (CXR), thoracic computed tomography (CT), and thoracic MRI. Lung volumes derived from CXR are based on the volumes contained within the thoracic contours.^6^, ^7^, ^8^, ^9^, ^10^ The major limitation of this approach is its compromised accuracy stemming from estimating a 3D phenomenon via 2D projection spaces and the difficulty in accurately decoupling the volumetric contribution of each lung due to anatomic overlap, especially in patients with severe thoracic deformities and spinal rotation. These issues become compounded for patients with deformed thorax and dynamic imaging.

Lung volumes derived from thoracic CT scans include tissue volumes and gas volumes^6^ within the segmented region. Comparable correlations have been observed between chest CT as compared with plethysmographic total lung capacity.^11^, ^12^, ^13^, ^14^ However, patients would have to be able to perform an adequate breath‐hold, which can be challenging for children. A serious issue with using CT in the pediatric setting, especially in the dynamic form, is its ionizing radiation. Spirometry measures the total volume of air in the lungs and all airway structures including the upper airways and not just the volume of air in lungs and it cannot separate the left and right thoracic components. 4D dynamic magnetic resonance imaging (dMRI) seems to be the only viable choice currently^15^ because of its soft tissue contrast, lack of ionizing radiation, and greater flexibility in the choice of imaging plane for acquisition. However, its major limitation is that it requires a relatively long acquisition time,^5^, ^15^, ^16^ as it can take up to 45 min to complete a dMRI study. Therefore, approaches to acquire the images more rapidly without sacrificing the accuracy of volumetric parameters estimation are highly desirable.

Although numerous static CXR, thoracic computed tomography,^17^, ^18^, ^19^ and MRI imaging techniques exist,^20^, ^21^, ^22^ with or without breath‐holding, they are not suitable for our goal of measuring respiratory dynamics from free‐breathing conditions, where humans spend most of their natural living time. Similarly, 2D techniques, even if dynamic,^23^, ^24^ and 3D static methods are not suited for our goal.

As such, we list some state‐of‐the‐art (SOTA) free‐breathing dynamic CT and dynamic MRI methods that are most pertinent to our goals. Several dynamic low‐dose CT methods are available.^23^, ^24^, ^25^, ^26^, ^27^, ^28^, ^29^ Some of them did not study normal pediatric subjects,^25^, ^26^, ^27^ some of them need breathing instructions, and some of them did no use auto‐segmentation methods.^26^, ^27^ One study assessed 4DCT of lung ventilation in children receiving radiation therapy.^29^ Photon‐counting CT (PCCT) is a new technique with advantages for pediatric imaging, enabling rapid scans with reduced motion artifact and need for sedation, and multi‐energy and ultra‐high‐resolution acquisition.^30^ Yet, we have not found literature on dynamic PCCT for studying lung function. Although dynamic low‐dose CT and PCCT are very attractive choices to use in some patients, for normal subjects (especially children), their use is discouraged given the ALARA principle.^31^ Moreover, it is very difficult to get approval from IRB and parents/guardians for use in normal pediatric subjects. For these reasons and to keep the modality the same for patients and normal subjects, we decided to focus on fast dMRI for fast image acquisition. Specifically, we present a novel sequence‐agnostic spatial sampling strategy, called *sparse dMRI*, or *s‐dMRI*, to significantly speed up a previously established dMRI scanning method for studying pediatric TIS.^5^, ^15^, ^16^, ^32^, ^33^, ^34^, ^35^, ^36^, ^37^ Distinct from advanced high‐end volumetric techniques (e.g., XD‐GRASP^38^ or iMoCo^39^) that often rely on specialized pulse sequences and intensive offline reconstruction, our framework utilizes standard, widely available 2D Cartesian sequences, making it immediately deployable on standard clinical scanners. Furthermore, while traditional slice‐sorting methods^40^ require dense full‐coverage imaging, the core contribution here is demonstrating that global multi‐organ dynamics can be accurately captured from extremely sparse spatial information (i.e., only 5 slices). A very preliminary version of this work was presented in the 2021 SPIE, the international society for optics and photonics, at its Medical Imaging conference proceedings.^41^ The present paper includes several new strategies for both optimal spatial and temporal sampling compared to just a basic method for spatial sampling, as well as comprehensive evaluation on retrospectively and prospectively acquired data sets compared to demonstration on just a small retrospective data set.

## 2 MATERIALS AND METHODS

### 2.1 dMRI scan protocol, subjects, image data

*Fully sampled scan*: For each sagittal slice location through the chest, image slices are acquired rapidly while the subject breaths freely, using the following imaging parameters: 3T‐MRI scanner (Verio, Siemens, Erlangen, Germany), balanced steady‐state free precession (bSSFP) sequence, TR = 4.28 ms, TE = 2.14 ms, voxel size ∼1×1×6 mm^3^, 320×320 matrix, bandwidth 258 Hz, and flip angle 53 degrees, FOV: 15 mm above apexes of lungs to base of kidneys. For each sagittal location, 80 slices are obtained over 8–14 breathing cycles at ∼480 ms/slice. Around 35 sagittal locations across the chest are imaged resulting in 2800–3200 2D slices. Total acquisition time is typically ∼45 min and up to an hour for larger subjects. From this set of slices, one representative 4D image comprising one full breathing cycle is constructed post hoc^15^, ^16^ from which volumetric parameters are derived to demonstrate the utility of dMRI in studying TIS.^5^, ^35^, ^36^, ^37^ The full scan data were acquired from 45 normal pediatric subjects, including 26 females and 19 males (average age 9.5 years, range 6.2–13.7). These 45 subjects overlap with 20 subjects in the preliminary paper.^41^ We realized that this full scan had temporal and possibly spatial redundancy for the purpose of computing dynamic volumes. We therefore studied ways to optimally sample these data spatio‐temporally so that the accuracy of volume estimation is not compromised but the acquisition time is reduced considerably.

*Sparse optimal scan*: In the sparse optimal protocol, 10 healthy subjects (4 males and 6 females with average age 16.3 years, range 13.1‐18.8) were scanned using the above sequence, with 5–7 sagittal slices uniformly located across each of right lung and left lung and 40 temporal slices for each sagittal location. These 10 subjects were also scanned at fully‐sampled protocol for comparison.

All datasets were acquired under an ongoing research protocol approved by the Institutional Review Board at the XXXXXX (anonymized) and XXXXXX (anonymized), along with Health Insurance Portability and Accountability Act authorization.

### 2.2 Overview of the framework

**FIGURE 1:**
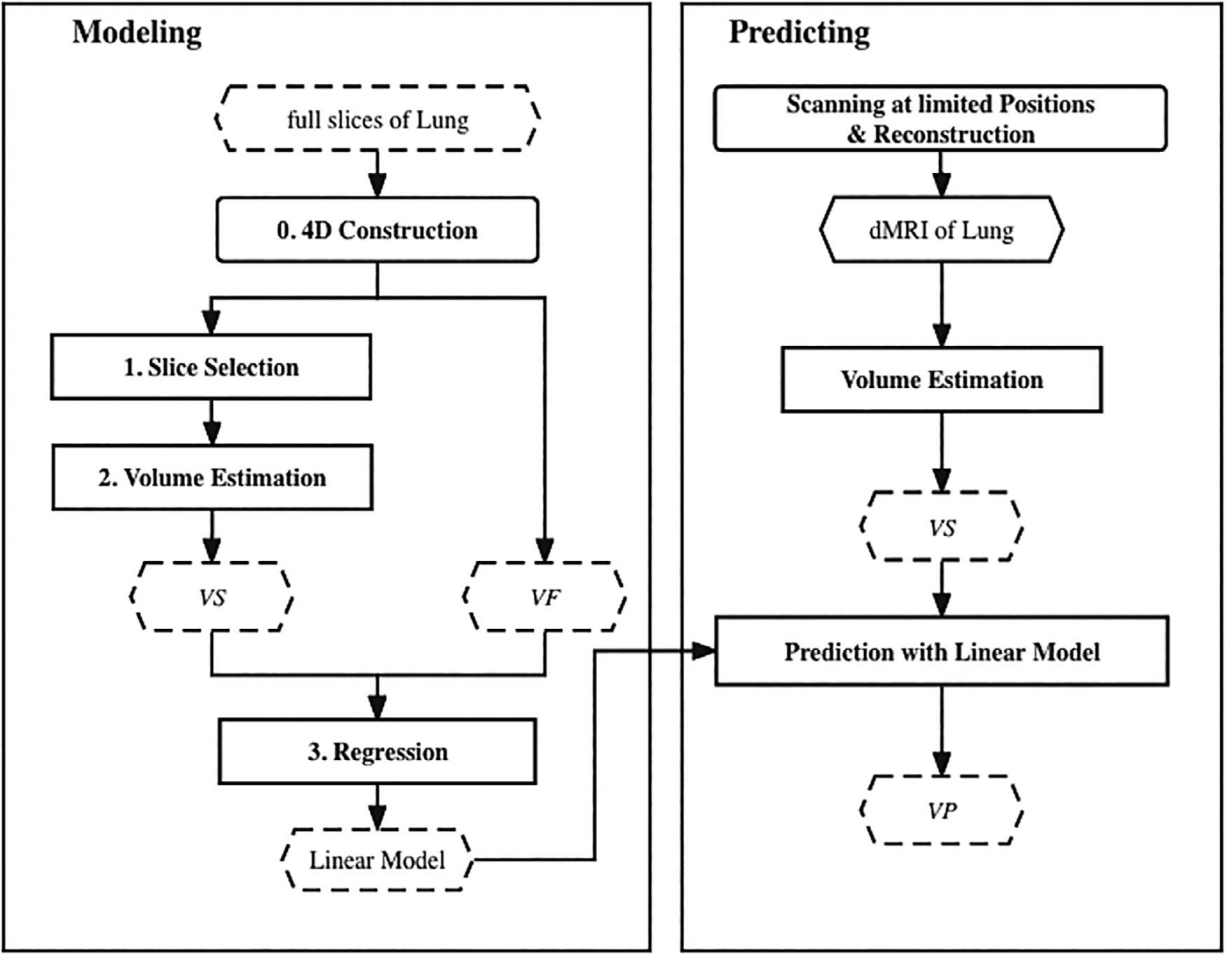
The framework of the proposed s‐dMRI method to estimate lung volumetric parameters. In the modeling stage, a linear regression model is created between sparse‐scan volume (*VS*) and full‐scan volume (*VF*) using training data. In the prediction stage, the predicted full volume (*VP*) from *VS* using the model and separate sparse scan data obtained either through simulation or via actual subject scans.

The s‐dMRI approach is depicted in Figure 1. In the modeling stage, we utilize 20 training dMRI datasets from *full scans*, simulate spatio‐temporal subsampling to create sparse scans from the full scans, compute various dynamic volumes, and obtain a regression model to represent the relationship between full‐scan volume (*VF*) and sparse‐scan volume (*VS*). In the prediction stage, we first compute *VS* from an independent cohort of sparse scans from 25 subjects, and then estimate the predicted full volume (*VP*) by using the prediction model. For details on the method, please see supplementary material.

### 2.3 Spatial subsampling

**FIGURE 2:**
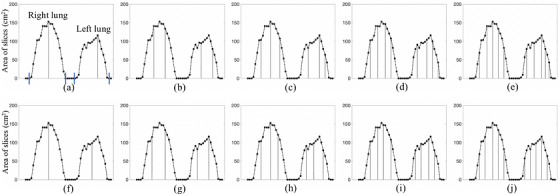
Illustration of the area curves for the two slice selection methods MAX and UFM. Sagittal locations are marked along the x‐axis, starting from the right side of the patient with the first sagittal location slightly outside the thorax and ending with the last slice outside the left side of the thorax. The medial and lateral edges of the right and left lungs are marked in (a). (a‐e) MAX: The numbers of selected slices are respectively from 1 to 5. (f‐j) UFM: The numbers of selected slices are respectively from 1 to 5. The continuous curves represent the lung area as a function of the slice location obtained from the slices in the full scan. The vertical bars denote the area of the lung in the slice selected at that location in the sparse scan.

**FIGURE 3:**
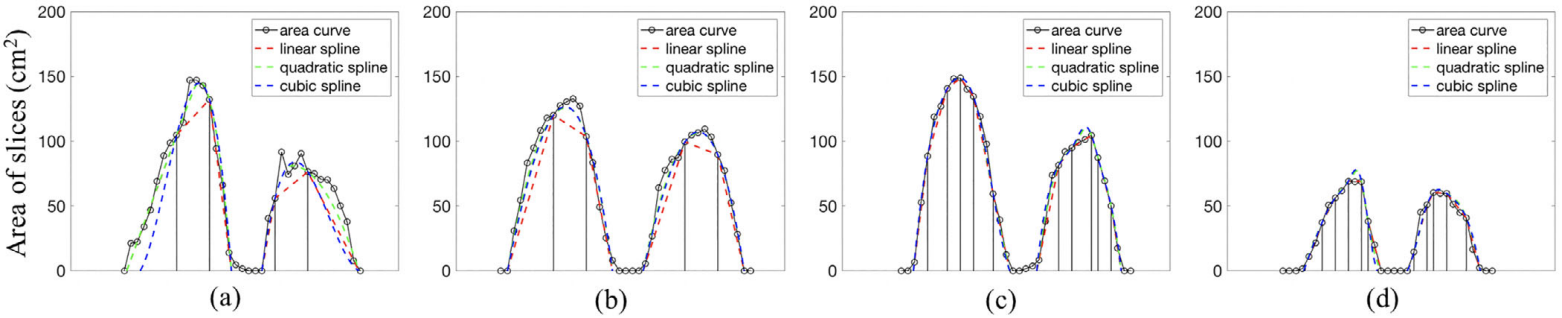
Illustration of the different interpolation methods on data sets from 4 different subjects. The layout is as in Figure 2. All examples use the MAX method of slice selection. Area curves shown: from full scan (black), LIN‐linear spline (red), QUA‐quadratic spline (green), and CUB‐cubic spline (blue). The number of selected slices is 2 in (a) and (b); 5 in (c) and (d).

There are three main parameters that determine optimal spatial subsampling: (1) Number of slices selected: The number of sagittal locations imaged in full scan (*N_full_*) varies from 15 to 22 slices between lateral and medial edges of each lung depending on body size. The idea of spatial subsampling is to select a smaller subset of slices (*N_sparse_*) between lateral and medial edges of each lung. (2) Method of selecting locations: We compared two strategies for selecting locations. In the uniform strategy UFM, *N_sparse_* locations uniformly situated between the edges of the lung are selected for imaging. In the MAX method, the sagittal location showing maximum lung cross‐sectional area is first selected for each lung. Subsequently, the medial and lateral regions separated by this anchor slice are sampled independently, with the remaining locations determined to be uniformly distributed within each region (Figure 2). Since UFM has no requirement of finding the location with the largest area, implementation of the method for actual clinical scanning is simple, practical, and straightforward. (3) Method of interpolation: A straightforward way of determining the sparse‐scan volume *VS* is via nearest neighbor (NN) interpolation. For a better approximation of the full‐scan volume *VF*, we compared three methods for interpolating the area curve to estimate *VS*—linear spline (LIN), quadratic spline (QUA), and cubic spline (CUB) (Figure 3).

To build the regression model, we utilized the full‐scan dMRI data and created sparse scans from them by selecting the sagittal locations. We designate the predicted lung volume derived from *VS* with the linear model as *VP* and use relative root mean squared (*rRMS*) error to evaluate the accuracy of the predictive model:

$$rRMS\;\left(N_{sparse}\right)=\sqrt{\frac{\sum_{i=1}^{N}{\left(\frac{VP\left(i\right)-VF\left(i\right)}{VF\left(i\right)}\right)}^{2}}{N}}\;,$$

where *N* represents the number of samples used for the regression process.

**FIGURE 4:**
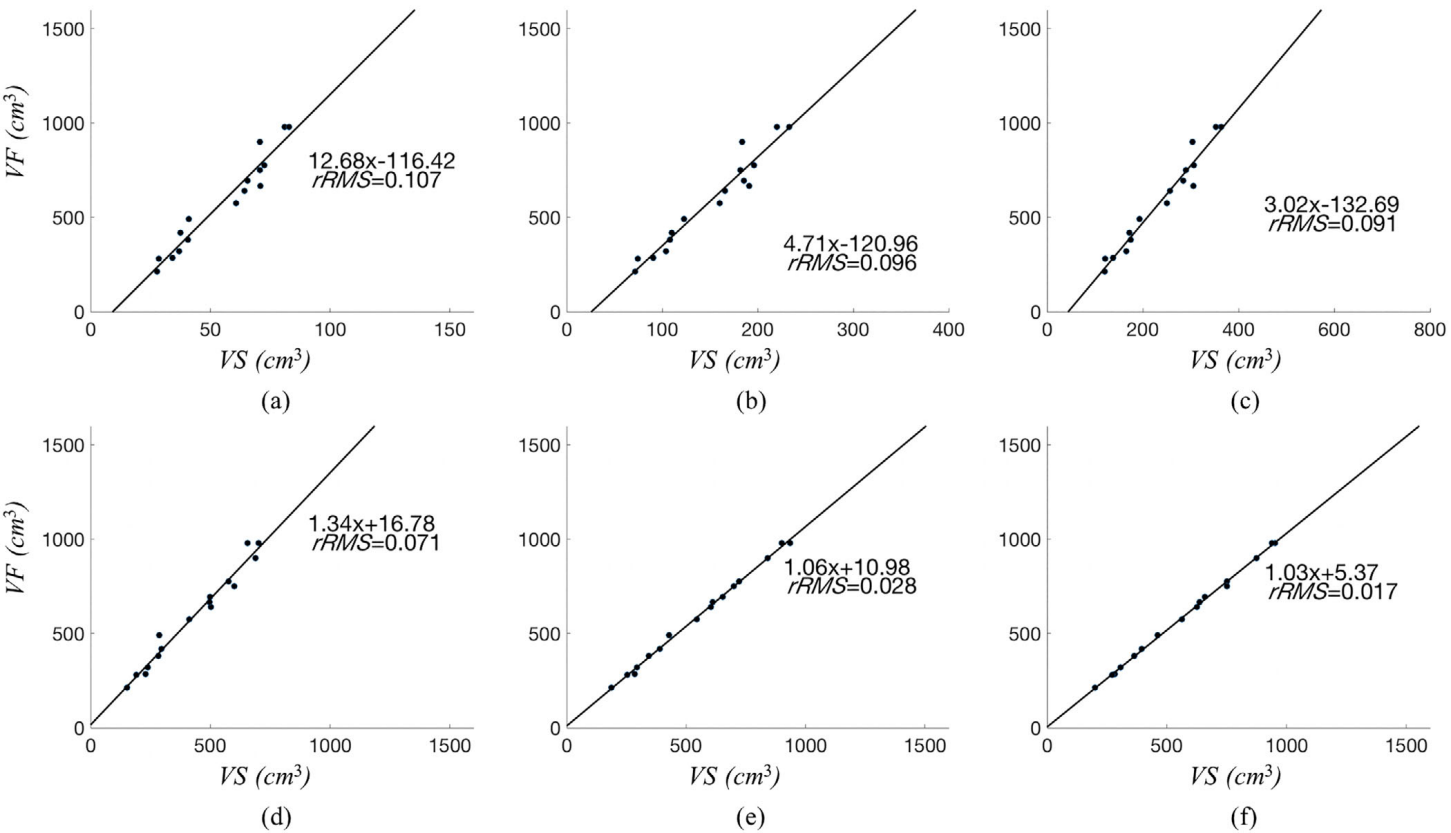
Illustration of linear regression from VS to VF. 20 samples of the left lung at end expiration from 20 normal subjects were used. The slice selection method used was UFM. 1st row: NN. 2nd row: LIN. (a), (d) Nsparse = 1; (b), (e) Nsparse = 3; (c), (f) Nsparse = 5. VF = Volume from Full scan. Nsparse = Number of Slices Selected.

Figure 4 illustrates the process of regression using UFM method with different *N_sparse_* and interpolation methods. Notably, refined interpolation methods shown in Row 2 achieve better regression accuracy than NN shown in Row 1. Clearly, accuracy improves as *N_sparse_* increases.

### 2.4 Temporal subsampling

The number of temporal slices acquired for each sagittal location in full scan is 80 time points, denoted as *T_full_*. To evaluate the impact of shortened scan durations, we simulated temporal subsampling by selecting the first *T_sparse_* time points from the full scan dataset. 4D construction was then performed using these temporally truncated sequences for different choices of *T_sparse_*, and the volume deviation from the full scan was determined as a function of the degree of temporal truncation.

## 3 RESULTS

### 3.1 Optimal parameter setting

The key independent measures we assess are right and left lung volumes at any time point such as end inspiration and end expiration, denoted, respectively, by *RLV_ei_*, *LLV_ei_*, *RLV_ee_*, and *LLV_ee_*. There are 4 parameters in the s‐dMRI approach: *N_sparse_*, slice selection methods (UFM or MAX), interpolation methods (NN, LIN, QUA, or CUB), and *T_sparse_*. Of these, *N_sparse_* and *T_sparse_* behave similarly—the larger the value, the more accurate will be the estimated volumes. They are also the arbiters of the time savings compared to the full scan. Slice selection methods and interpolation methods determine the accuracy of approximation achieved for fixed values of *N_sparse_* and *T_sparse_*. Therefore, for different possible choices of *N_sparse_* and *T_sparse_*, we systematically study the tradeoff between accuracy and efficiency to choose the best setting for the parameters.

**FIGURE 5:**
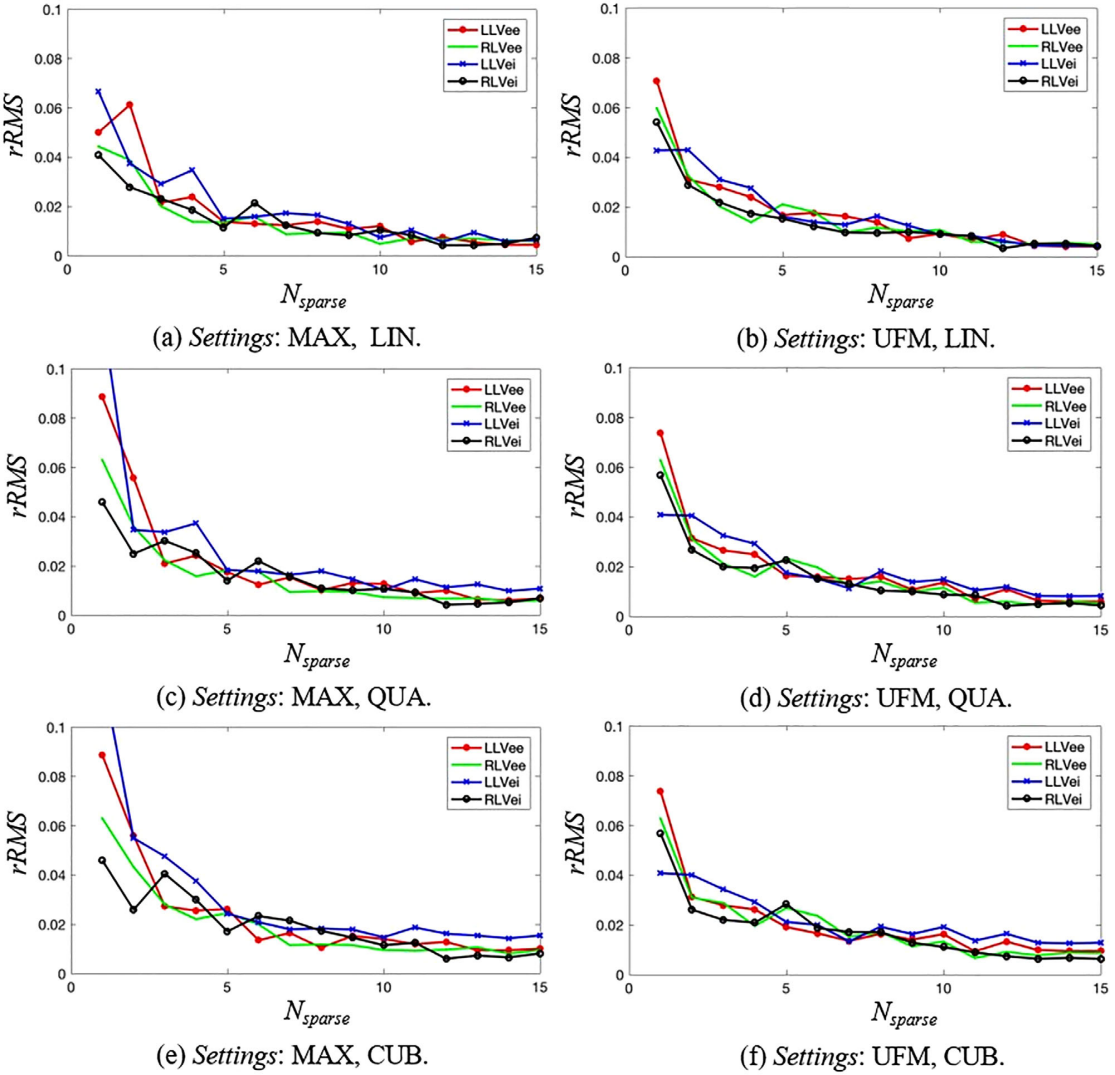
Illustration of relative root mean squared (*rRMS*) error of model fit for the 4 static lung volumes *RLV_ei_*, *LLV_ei_*, *RLV_ee_*, and *LLV_ee_* as a function of the parameters *N_sparse_* in the modeling stage.

Figure 5 illustrates the variation of *rRMS* in spatial subsampling as a function of *N_sparse_* with different slice selection methods and interpolation methods on the 20 data sets used for modeling for the four volumes *RLV_ei_*, *LLV_ei_*, *RLV_ee_*, and *LLV_ee_*. Since NN is non‐competitive, it is excluded from consideration. Notably, for *N_sparse_* ≥ 5, *rRMS* hovers around or falls below 2%, with LIN having a slight advantage over other interpolation methods. We may also note that there is not much difference in *rRMS* errors between slice selection methods MAX and UFM for *N_sparse_* ≥ 5. Since UFM is simpler to actually implement in performing sparse scans, we recommend UFM as the method of slice selection.

Since *N_sparse_* = 5 already achieves very low error, the changes we observe in volumes from pre‐ to post‐operative condition in TIS treatment are typically greater than 20%,^7^ and increasing *N_sparse_* beyond 5 lowers error insignificantly and gradually, for spatial subsampling we recommend the choice of *N_sparse_* = 5, UFM, and LIN, at least for the TIS application.

**FIGURE 6:**
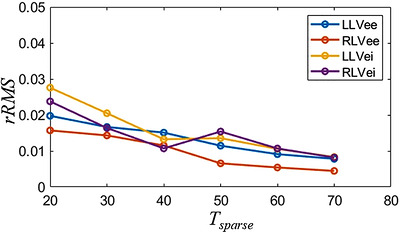
Illustration of relative root mean squared (*rRMS*) error of model fit for the 4 volumes *RLV_ei_*, *LLV_ei_*, *RLV_ee_*, and *LLV_ee_* and as a function of the parameter *T_sparse_* in the modeling stage.

Figure 6 illustrates the variation in *rRMS* in temporal subsampling as a function of *T_sparse_* on the 20 data sets used for modeling. Table 1 summarizes the actual *rRMS* values. Recall that for each value of *T_sparse_*, full spatial volume is considered without any spatial subsampling to study the effect of temporal sub‐sampling on its own.

**TABLE 1:** *rRMS* errors in volume estimation for different choices of the number of temporal samples *T_sparse_* and no spatial subsampling.

*T_sparse_*	20	30	40	50	60	70
LLVee	0.020	0.017	0.015	0.011	0.009	0.008
RLVee	0.016	0.014	0.012	0.007	0.005	0.004
LLVei	0.028	0.020	0.013	0.014	0.011	0.008
RLVei	0.024	0.016	0.011	0.015	0.011	0.008

Abbreviations: *LLV_ee_*, Left Lung Volume at End‐Expiration; *LLV_ei_*, Left Lung Volume at End‐Inspiration; *RLV_ee_*, Right Lung Volume at End‐Expiration; *RLV_ei_*, Right Lung Volume at End‐Inspiration; *rRMS*, relative Root Mean Squared error.

Notably, increasing *T_sparse_* from 40 to 70 provides diminishing improvements in accuracy at the expense of prolonged scan time, whereas reducing it below 40 severely compromises volume estimation. Therefore, *T_sparse_* = 40 is selected as the optimal baseline configuration to balance performance and efficiency.∖

In the actual analysis of the accuracy of the whole s‐dMRI process presented below, we will use the optimal settings of UFM, LIN, and *T_sparse_* = 40.

### 3.2 Assessing accuracy of the s‐dMRI process

Using the optimal settings obtained as above, we determine the actual volume prediction accuracy in terms of *rRMS* error from retrospectively obtained data subsampled from full dMRI scans as well as prospectively acquired actual sparse scans.

#### 3.2.1 Retrospective analysis

**FIGURE 7:**
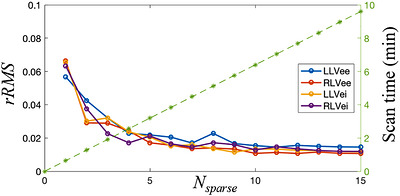
Illustration of relative root mean squared (*rRMS*) error of the s‐dMRI process for estimating the 4 key lung volumes *RLV_ei_*, *LLV_ei_*, *RLV_ee_*, and *LLV_ee_* as a function of the parameter *N_sparse_*. Errors are estimated from sparse scans simulated from full scans of 25 normal subjects. The curve of mean acquisition time per lung is also shown as a dashed curve. *LLV_ee_* = Left Lung Volume at end‐expiration, *LLV_ei_* = Left Lung Volume at end‐inspiration, *N_sparse_* = Number of Slices Selected. *RLV_ei_* = Right Lung Volume at end‐inspiration. *RLV_ee_* = Right Lung Volume at end‐expiration.

Figure 7 summarizes s‐dMRI predictive performance in terms of *rRMS* error on the 25 test subjects for the 4 volume measures for the parameter setting of UFM, LIN, and *T_sparse_* = 40 for different values of *N_sparse_*. We also report the mean and standard deviations across the test subjects in Table 2. For this analysis, the sparse scans were obtained via simulation from the full scans. The mean acquisition time per lung is also shown in Figure 7 corresponding to different values of *N_sparse_* as a dashed curve. The upper right corner of the curve represents the actual acquisition time per lung that was measured for *T_sparse_* = 40 without leaving out sagittal locations and corresponding to roughly *N_sparse_* = 15. In other words, the scan corresponding to that point involved temporal but not spatial sub‐sampling. For lower values of *N_sparse_*, the acquisition time shown is an estimation based on the number of slices imaged per lung. Notably, only with temporal sub‐sampling (*T_sparse_* = 40) and without any spatial sub‐sampling, the total acquisition time can be reduced from ∼45 min to ∼20 min with an *rRMS* error of under 0.4% for the 4 key volume parameters compared to the full scan!

**TABLE 2:** Mean and Standard deviation of relative root mean squared (*rRMS*) errors for different choices of number of slices in the sparse scan *N_sparse_*. Here, method of selecting locations = UFM (uniform) and method of interpolation = LIN.

*N_sparse_*	*LLV_ee_*	*RLV_ee_*	*LLV_ei_*	*RLV_ei_*
1	0.0418 ± 0.0392	0.0544 ± 0.0387	0.0453 ± 0.0483	0.0475 ± 0.0428
2	0.0341 ± 0.0258	0.0221 ± 0.0193	0.0249 ± 0.0175	0.0302 ± 0.0227
3	0.025 ± 0.0204	0.0237 ± 0.0168	0.0269 ± 0.0178	0.0179 ± 0.014
4	0.0193 ± 0.0124	0.0208 ± 0.0127	0.0209 ± 0.0114	0.0149 ± 0.0086
5	0.0182 ± 0.0124	0.0153 ± 0.0078	0.0162 ± 0.0127	0.0174 ± 0.0124
6	0.0184 ± 0.0093	0.0129 ± 0.0091	0.0132 ± 0.0076	0.0134 ± 0.0098
7	0.0137 ± 0.0102	0.0112 ± 0.008	0.0132 ± 0.0091	0.0117 ± 0.0088
8	0.0187 ± 0.0133	0.011 ± 0.0088	0.0113 ± 0.0079	0.0142 ± 0.0098
9	0.0144 ± 0.0085	0.0098 ± 0.0093	0.0091 ± 0.0072	0.0134 ± 0.0089
10	0.0118 ± 0.0102	0.0086 ± 0.0067	0.0096 ± 0.0095	0.0103 ± 0.0079
11	0.0111 ± 0.0096	0.0095 ± 0.0063	0.0098 ± 0.0092	0.0118 ± 0.009
12	0.0124 ± 0.0096	0.0082 ± 0.007	0.0097 ± 0.0082	0.0108 ± 0.0082
13	0.0113 ± 0.0101	0.0085 ± 0.008	0.0102 ± 0.0073	0.0098 ± 0.008
14	0.0112 ± 0.0097	0.0081 ± 0.0073	0.0094 ± 0.0075	0.0094 ± 0.0076
15	0.0111 ± 0.0096	0.0081 ± 0.0072	0.0092 ± 0.0074	0.0091 ± 0.0078

Abbreviations: *LLV_ee_*: Left Lung Volume at End‐Expiration; LL*V_ei_*: Left Lung Volume at End‐Inspiration; *RLV_ee_*: Right Lung Volume at End‐Expiration; *RLV_ei_*: Right Lung Volume at End‐Inspiration

#### 3.2.2 Prospective analysis

For the prospectively acquired sparse scans (*N_sparse_* = 5–7, *T_sparse_* = 40), the *rRMS* errors in the different volume measures are as listed in Table 3. The reference measures used in this evaluation were the volumes obtained from the fully‐sampled scans of the same subjects. The mean acquisition time for the sparse scans was 4.5 min for each lung and the total acquisition time was ∼9 min. The error and the acquisition time are comparable to those from retrospective simulated scans for *N_sparse_* = 5 and *T_sparse_* = 40.

**TABLE 3:** *rRMS* errors in volume measures in our prospective study involving sparse scans.

*LLV_ee_*	*RLV_ee_*	*LLV_ei_*	*RLV_ei_*
0.0528	0.0505	0.0464	0.0512

Abbreviations: *LLV_ee_*, Left Lung Volume at End‐Expiration*; LLV_ei_*, Left Lung Volume at End‐Inspiration*; RLV_ee_*, Right Lung Volume at End‐Expiration*; RLV_ei_*, Right Lung Volume at End‐Inspiration*; rRMS*, relative Root Mean Squared error.

**FIGURE 8:**
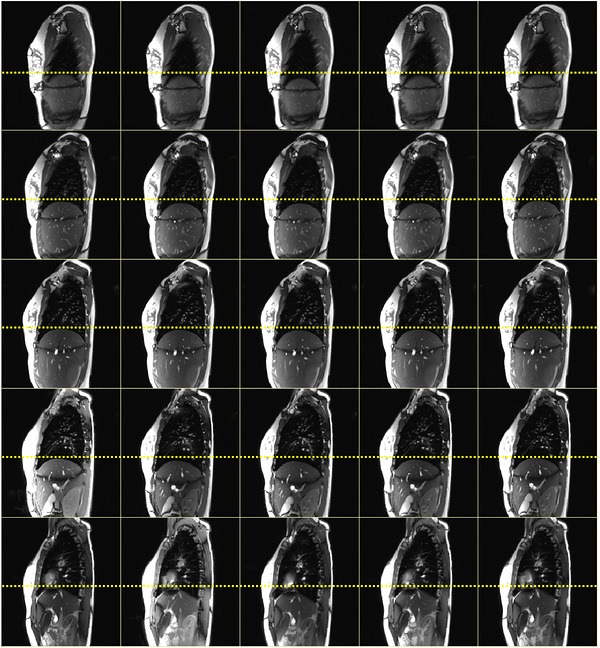
An example of the 4D constructed image of our sparse scan covering the right lung of a normal 17.6 years old female subject. Each row shows one respiratory cycle of 5 time points for one sagittal location. The different rows indicate 5 different sagittal locations. The yellow dashed line represents the end‐expiration level of the diaphragm dome.

In Figure 8, we display the 2D spatial and temporal slices corresponding to the right lung selected from the 4D image constructed from a sparse scan of a normal 17.6 years old female subject. Note that the images shown here are the original, sparsely acquired slices without any spatial interpolation.

## 4 DISCUSSION

In this paper, we presented a novel approach using limited‐slice, short scan‐time, free‐breathing, thoracic dynamic MRI to accurately estimate key lung dynamic volumetric parameters. Taking a well‐established fully‐sampled scan that takes 45 min – 1 h per scan as reference, we demonstrated a root mean squared error in estimating volumes of less than 2% in retrospective simulations and approximately 5% in prospective assessments for pediatric subjects with a total acquisition time of ∼9 min by utilizing only 5 sagittal MRI slices through each lung and for each sagittal slice location acquiring 40 slices as a time sequence under free breathing conditions covering roughly 5–10 pediatric respiratory cycles. This slight increase in prospective error is primarily driven by the independent nature of the separate scan sessions, where subtle variations in the patient's baseline breathing volume and minor anatomical positioning shifts introduce an inherent inter‐scan misalignment rather than algorithmic instability. The method exploits the smoothness of the lung area in sagittal slices as a function of the slice location (Figures 2 and 3) for each lung to achieve reduced acquisition time. By scanning at full spatial sampling (i.e., *N_sparse_* = *N_full_*) but reducing temporal samples by half (i.e., *T_sparse_* = 0.5 *T_full_*), we showed that the above error drops to ∼0.4% with a total acquisition time of ∼20 min. As such, the proposed s‐dMRI approach constitutes a practical approach to measure dynamic volumes and their changes separately for each lung in any application that needs to study the dynamic function of the two lungs separately under natural free‐breathing conditions. The approach can lead to increased patient comfort and convenience for practical real‐world clinical applications, as we have observed in the TIS application. This may also potentially lead to improved image quality and usability due to a reduction of patient motion, discomfort, and abnormal breathing patterns.

In the TIS application, the pre‐ to post‐operative changes in the key volume parameters and tidal volumes are > 20% and > 40%, respectively,^5^ as such the above errors are unlikely to interfere with our ability to measure operative treatment response. Even the more accurate version with *N_sparse_* = *N_full_* and *T_sparse_* = 40 is highly practical with a 20‐min acquisition time.

Dynamic MRI methods in the literature that demonstrated full 4D imaging capability under free‐breathing conditions with demonstrated acquisition time practicality and accuracy metrics for measuring lung dynamic volumes in pediatric subjects are few. Table 4 provides a comparative analysis of the proposed approach and existing methods in the literature for dynamic lung volume estimation. Most existing techniques require breath maneuvering such as breath holding or separate navigator scanning or focus on analyzing the motion of the diaphragm only, often at a few selected points or slice locations, or for achieving adequate spatio‐temporal resolution employ smaller field of view that does not cover the abdominal organs. Their conversion to estimate volumes routinely is not obvious or face additional challenges. In other words, a practical method for pediatric use without radiation concerns that can perform scanning in 10–20 min for estimating free‐breathing dynamic volumes and tidal volumes separately for each lung does not seem to exist at present.

**TABLE 4:** Summary of related papers from literature.

	Modality	Dimension	Breathing	Acquisition time	Population	# of subjects tested	Voxel size (mm^3^)
Qanadli et al., 1999^44^	MRI	2D	Breath hold	14s per slice	Adults (24–32)	15	4.6 × 4.6 × 10
Coxson et al., 2008^45^	CT	3D	Breath hold	Not available	Adults	57	1.25 × 1.25 × 10
Fuld et al., 2012^46^	CT	3D	Breath hold	< 10 s	Adults (20–64)	37	0.61 × 0.61 × 0.5
Jahani et al., 2015^47^	CT	4D	Breath hold	∼ 1 min	Adults	6	0.5 × 0.5 × 0.75
Higano et al., 2017^40^	3D radial UTE MRI	3D	Free breathing	9–16 min	Adults & neonates	22	0.78 × 0.78 × 0.78
Martini et al., 2019^48^	dMRI	2D	Free breathing	2 resp cycles	Adults	39	NA
Safavi et al., 2020^49^	MRI	2D	Breath hold	10 s per slice	Adults (> 18 years)	31	NAxNAx6
Sato et al., 2022^50^	dMRI	2D	Free breathing	121 ms per image	Not available	31	2 × 2 × 13.5
XD‐GRASP^38^	MRI	3D	Free breathing	∼95s/partition x 80 partitions	Adult	5	1.5 × 1.5 × 6
iMoCo^39^	MRI	3D	Free breathing	5min30s	Adult Pediatric	11	1.1 × 1.1 × 1.1

Abbreviation: NA, not available.

Key competing methods are,^40^ which reports an ultra‐short echo‐time MRI, and XD‐GRASP^38^ and iMoCo^39^ methods that provide adequate spatiotemporal resolution with about 5–10 min of acquisition time. While these methods excel in high‐resolution localized anatomical scanning, capturing individual non‐linear respiratory motion trajectories across a wide field of view within constrained scan times remains challenging. In our application, and hence in the full scan and sparse scan, the field of view extends to the inferior aspect of the kidneys since we are interested in studying how respiratory restrictions affect the mobility of key abdominal organs like the liver, kidneys, and spleen and vice versa, as already demonstrated,^15^, ^16^, ^36^, ^37^, ^42^, ^43^ which utilized the same acquisition protocol. As such image quality and coverage should afford accurate auto‐segmentation of not just the lungs but also these key organs. Studying the utility of such recent new imaging sequences for our purposes to reduce the acquisition time for each slice in our method but then use our current paradigm of weaving the selected slices together ingeniously through the OFx strategy^16^ to form a 4D image will be one of our future goals.

To indicate the range of acquisition time and the potential for its improvement, we consider two extreme cases: small and large pediatric subjects with chest widths of 12 and 36 cm, respectively. Assume that the number of temporal slices collected per sagittal position is *T_sparse_* = 40 based on the optimal temporal sampling found in our approach. There are 2 other variables that are critical to optimizing acquisition time and image quality: tps = data acquisition time per slice and *N_sparse_ *= number of sagittal positions imaged which we assume to equal *N_full_*. For small subjects *N_sparse_ *= *N_full_* will be roughly 20 with the 6 mm slice spacing in our current protocol, and the total acquisition time will be ∼7 min with the current time per slice tps = 480 ms, and for large subjects, acquisition time will be ∼20 min. In the future, by using the same imaging sequence but faster techniques of data collection and image reconstruction such as via compressed sensing and parallel imaging, we expect to reduce tps considerably. Although this may call for increasing *T_sparse_* to make sure that data for enough respiratory cycles are gathered for each sagittal location, we expect to reduce acquisition time even for large subjects to ∼4 min, as indicated by our recent very preliminary studies (unpublished).

The main limitation of our study is the somewhat small sample size and the lack of demonstration of the s‐dMRI approach in adults for broader applicability. Fortunately, since the respiratory rate is much lower in adults, we may be able to reduce *T_sparse_* further to reduce acquisition time. However, it remains to be investigated if we may need to also increase *N_sparse_* beyond 5, since the body width is greater in adults than in children, which may prolong the acquisition time.

## 5 CONCLUSION

In conclusion, s‐dMRI offers a practical approach for computing dynamic volumes of individual lungs accurately that can be used routinely with no radiation concern, especially on patients who cannot tolerate long acquisition times. The s‐dMRI sequence employed in this paper is a general sequence and can be easily implemented on any modern MRI scanner.

## CONFLICT OF INTEREST STATEMENT

The authors declare no conflicts of interest.

## Supporting information



Supporting Information
